# *In vitro* and *in vivo* analysis of antimicrobial agents alone and in combination against multi-drug resistant *Acinetobacter baumannii*

**DOI:** 10.3389/fmicb.2015.00507

**Published:** 2015-05-27

**Authors:** Songzhe He, Hui He, Yi Chen, Yueming Chen, Wei Wang, Daojun Yu

**Affiliations:** ^1^The Affiliated First Hospital of Hangzhou, Zhejiang Chinese Medical UniversityHangzhou, China; ^2^Department of Clinical Laboratories, Hangzhou First People's HospitalHangzhou, China

**Keywords:** *Acinetobacter baumannii*, multi-drug resistant, ultrasonic atomization, pneumonia infection model, combination treatment

## Abstract

**Objective:** To investigate the *in vitro* and *in vivo* antibacterial activities of tigecycline and other 13 common antimicrobial agents, alone or in combination, against multi-drug resistant *Acinetobacter baumannii*.

**Methods:** An *in vitro* susceptibility test of 101 *A. baumannii* was used to detect minimal inhibitory concentrations (MICs). A mouse lung infection model of multi-drug resistant *A. baumannii*, established by the ultrasonic atomization method, was used to define *in vivo* antimicrobial activities.

**Results:** Multi-drug resistant *A. baumannii* showed high sensitivity to tigecycline (98% inhibition), polymyxin B (78.2% inhibition), and minocycline (74.2% inhibition). However, the use of these antimicrobial agents in combination with other antimicrobial agents produced synergistic or additive effects. *In vivo* data showed that white blood cell (WBC) counts in drug combination groups C (minocycline + amikacin) and D (minocycline + rifampicin) were significantly higher than in groups A (tigecycline) and B (polymyxin B) (*P* < 0.05), after administration of the drugs 24 h post-infection. Lung tissue inflammation gradually increased in the model group during the first 24 h after ultrasonic atomization infection; vasodilation, congestion with hemorrhage were observed 48 h post infection. After 3 days of anti-infective therapy in groups A, B, C, and D, lung tissue inflammation in each group gradually recovered with clear structures. The mortality rates in drug combination groups(groups C and D) were much lower than in groups A and B.

**Conclusion:** The combination of minocycline with either rifampicin or amikacin is more effective against multi-drug resistant *A. baumannii* than single-agent tigecycline or polymyxin B. In addition, the mouse lung infection by ultrasonic atomization is a suitable model for drug screening and analysis of infection mechanism.

## Introduction

*Acinetobacter baumannii* is a nonfermentative, gram-negative bacillus, whose natural reservoir still remains to be determined. It can represent an opportunistic pathogen in humans, and often causes nosocomial infections in immunocompromised patients, such as pneumonia, urinary tract infection, and sepsis (Dettori et al., 2014). Until recently, most studies on *A. baumannii* have focused on antibiotic resistance, treatment and epidemiological analysis (Erac et al., 2014). With the large amount of clinical applications of antibiotics, the isolation rate of drug-resistant *A. baumannii* has been gradually rising, and the emergence of multi-drug resistant strains poses a big challenge for antibiotic treatment (Lee et al., 2011; Sievert et al., 2013). In recent years, the drug resistance issue has attracted worldwide attention. New therapeutic strategies against *A. baumannii* are urgently needed.

The treatment choices available for this infection are limited. Tigecycline and polymyxin B have shown some efficacy, as evidenced by both *in vitro* and *in vivo* experiments (Durante-Mangoni et al., 2013; Stein and Babinchak, 2013; Chuang et al., 2014). However, due to the lack of large-scale clinical studies, as well as the high cost of tigecycline and the potential nephrotoxic effects of polymyxin, the clinical use of these drugs has been limited. Combinations of two or more antimicrobial drugs are often used for the treatment of multi-drug resistant *A. baumannii* infections. It has been reported that meropenem, polymyxin B and minocycline have synergistic effects *in vitro* against *A. baumannii* (Zusman et al., 2013; Ning et al., 2014). In addition, the combination of sulbactam with imipenem displays synergistic bactericidal activity in the lung tissue (Dinc et al., 2013). The combination of rifampicin with imipenem, sulbactam, and colistin has the ability to potentiate the anti-infection activity of these drugs (Pachón-Ibáñez et al., 2010).

Although both *in vitro* and *in vivo* data support the efficacy of certain antibiotics against *A. baumannii*, discrepancies have been found in the results, due to the unstable or inefficient animal model (Mutlu Yilmaz et al., 2012). Hence, it is of particular importance to establish a stable model for drug screening or for investigating infection mechanisms. At present, the mouse model of *A. baumannii* infection has been shown not to be successful, as only a self-limiting bacterial pneumonia is induced, even if a high dose of bacteria is administered. To improve this model, some research groups used immunocompromised mice or mucin-treated mice to increase their sensitivity to *A. baumannii* (van Faassen et al., 2007; Pichardo et al., 2010). Lung infections in murine models have been produced by direct tracheotomy infection (Eveillard et al., 2010), micro-tracheal injection (Eveillard et al., 2010), or intranasal administration (Russo et al., 2008). However, those methods have clear disadvantages, leading to low infection rate (Qiu et al., 2009).

Given the low infection rate and instability of the current pneumonia models, we intended to establish a new *A. baumannii* infected mouse model using ultrasonic atomization. The drugs that were found to have antibacterial effect *in vitro*, tigecycline and polymyxin B were validated in this *in vivo* model. Additionally, some relatively cost-effective antibiotics were compared, including amikacin, minocycline and rifampicin, and the efficacy of the combinations including those drugs was analyzed. In conclusion, this study presented a new *in vivo* model for future studies, and provided experimental evidence of an effective combination therapy for multi-drug resistant *A. baumannii* infection.

## Materials and methods

### Strains

One hundred and one multi-drug resistant *A. baumannii* strains were obtained from Hangzhou First People's Hospital and were identified by the Vitek 2 Compact analyzer (BioMérieux SA, France). Multi-drug resistant strains were identified by drug susceptibility test and stored at −80°C. *Pseudomonas aeruginosa* ATCC27853 were used as control strain.

### Experimental animals

Five hundred specific pathogen-free BALB/c mice (half male and half female, weight 12–14 g, age 4 weeks) were bred at a temperature of 18–25°C and humidity of 40–70%. The license number was SCXK (Shanghai) 2013–0016. According to “Animal Quality Management Approach” (1997), the experimental procedures were under the approval by the Experimental Animal Center of Zhejiang Chinese Medical University.

### Experimental drugs and main instruments

The drugs purchased and used in this experiments were the following: imipenem/cilastatin sodium (IMP/CS) (Merek sharp & Dohme Corp., New Jersey, United States); piperacillin/tazobactam sodium (TZP) (Wyeth Lederle SPA, New Jersey, United States); cefoperazone/sulbactam sodium (SCF) (Pfizer, New York, United States); ceftazidime (CAZ) (Hailing Chemical Pharmaceutical Co., Ltd., Haikou, Hainan); rifampicin (RIF) (Shuangding Pharmaceutical Co., Ltd., Shenyang, Liaoning); amikacin (AMK) (Qilu Pharmaceutical Co., Ltd., Jinan, Shandong); levofloxacin (LEV) (Yangtze River Pharmaceutical Group Ltd., Taizhou, Jiangsu); polymyxin B (PB) (Japan Pharmaceutical Industry Co., Ltd., Taipei, Taiwan); tigecycline (TIG) (Hisun Pharmaceutical Co., Ltd., Taizhou, Zhejiang); minocycline (MNO) (Wyeth Pharmaceutical Co., Ltd., Suzhou, Jiangsu); chloramphenicol (C) (Modern Pharmaceutical Co., Ltd., Shanghai); erythromycin (E) (Kelun Pharmaceutical Co., Ltd., Chengdu, Sichuan); fosfomycin sodium (FOS) (Northeast Pharmaceutical Group Shenyang No.1 Pharmaceutical Co., Ltd., Shenyang, Liaoning); methotrexate (MTX) (Hengrui Pharmace utical Co., Ltd., Lianyungang, Jiangsu). Ultrasonic Nebulizer (402A1 type, Jiangsu Diving Medical Equipment Co., Ltd., Suzhou, Jiangsu).

### Minimum inhibitory concentration (MIC)

According to standard Regulations of Clinical Laboratory (Piewngam and Kiratisin, 2014), the broth dilution method was used to detect MIC. Briefly, solutions with different concentrations of antimicrobial agents were added to a sterile 96-well polystyrene plate. A concentration of 0.5 McFarland units (5 × 10^8^ CFU/ml) of bacterial suspension was diluted with Lysogeny Broth (LB) (final concentration 5 × 10^5^ CFU/ml) and was added to each well of the plate. The plate was sealed and incubated at 35°C for 18–24 h. *Pseudomonas aeruginosa* ATCC27853 was used as control. The concentration of the drugs that completely inhibited bacterial growth was defined as MIC. The evaluation of tigecycline was based on FDA standards and the other antibiotics were in accordance with Clinical and Laboratory Standards Institute (2014).

### Chequerboard assay

The drug combination regimens are listed in Table 1. A micro-dilution method associated with checkerboard was applied in the drug combination screening on three randomly selected strains. Drug interactions were determined by the fractional inhibitory concentration index (FICI). FICI was defined as FICI = MIC_A2_/MIC_A1_+MIC_B2_/MIC_B1_ and FICI index ≤0.5, 0.5–1, 1–4, >4 were used to define synergism, addition, non-relation or antagonism, respectively (Sopirala et al., 2010).

**Table 1:** **The scheme for antibiotics combination**.

**Antibiotics**	**RIF**	**TZP**	**C**	**FOS**	**E**	**MNO**	**AMK**	**IMP/CS**	**PB**
PB	+	+	+	+	+	−	−	−	−
TIG	+	−	+	+	+	+	+	−	−
MNO	+	−	+	+	+	−	+	−	+
FOS	−	−	+	−	+	−	+	+	−
C	−	−	−	−	−	−	−	+	−

*PB, Polymyxin B; TIG, Tigecycline; MNO, Minocycline; FOS, Fosfomycin sodium; C, Chloramphenicol; RIF, Rifampicin; TZP, Piperacillin/tazobactam sodium; E, Erythromycin; IMP/CS, Imipenem/cilastatin sodium; AMK, Amikacin; “+” represents the combination of two drugs; “−” represents the non-combination of two drugs*.

### Time-kill curve experiments

Tigecycline, polymyxin B, minocycline, rifampicin, chloramphenicol and fosfomycin sodium were used in the time-kill curve experiments. Drug concentrations of 0.5 × MIC, 1 × MIC, 2 × MIC, and 4 × MIC were chosen for these experiments. Briefly, tubes containing LB with antibiotics were inoculated with *A. baumannii* in a log-phase inoculum of roughly 5 × 10^5^ CFU/ml. Tubes were incubated in an ambient atmosphere at 35°C. At time 0, 2, 4, 8, and 24 h after inoculation, serial 10-fold dilutions were performed and aliquots were plated onto nutrient agar. The time-kill curve experiments were performed twice and results were analyzed by mean colony count values from the duplicate plates for each isolate (Rodriguez et al., 2010). The bactericidal activity of single antibiotics or combinations was defined as ≥3 log_10_ CFU/ml decrease in the viable count compared with the initial inoculum. Synergism and antagonism were respectively defined as ≥2 log_10_ CFU/ml decrease or increase in the viable count with the combination compared with the most active agent alone at different time points (Tängdén et al., 2014).

### Establishment of pneumonia model and drug treatment

After 1 week adaptation, the median lethal dose of methotrexate was detected (data not shown). The experiment included control (10 mice), model (90 mice) and treatment groups (divided into A, B, C, and D group, 80 mice per group). The mice in the control group were fed normally, while those in the model and treatment groups received an intraperitoneal injection of methotrexate (0.3 mg/day) for 3 consecutive days. The dose of methotrexate was calculated based on body surface area (Men and Mice medication ratio is 1:0.0026). Three days later, the mice in the model and treatment groups were given with 10% chloral hydrate (250 mg/kg) by intraperitoneal injection. The anesthetized mice were placed in a plastic container with two ports. A concentration of 5 × 10^8^ CFU/ml of multi-drug resistant *A. baumannii* was placed in a small ultrasonic nebulizer. *A. baumannii* flowed into the plastic container through an entry, and was discharged from the other end. In total, the ultrasonic frequency was 1.7 MHz ± 10%, atomization speed was 2 ml/min and the time of continuous atomization was 30 min. All operations were performed in a biological safety cabinet.

After infection with multi-drug resistant *A. baumannii*, the mice were randomly assigned to one of the following treatment groups: tigecycline (group A), polymyxin B (group B), minocycline + amikacin (group C), minocycline + rifampicin (group D). Mice within each group were also randomly assigned to four sub-groups of different treatment time (post infection 0, 4, 24, and 48 h), each sub-group was 20 mice (10 mice for recording the body symptoms and mortality, 10 mice for detection on counts of white blood cells and pathological examination of lung). Antimicrobial agents were given by intraperitoneal injection, with dosages as follows: group A (tigecycline, 10 mg/kg, q12h), group B (polymyxin B, 5 mg/kg, q6h), group C (minocycline, 7.5 mg/kg, q12h and amikacin, 7.5 mg/kg, q12h), and group D (minocycline, 7.5 mg/kg, q12h and rifampicin, 25 mg/kg, per day). These doses were chosen according to previous pharmacokinetic and pharmacodynamic data from experimental models (Song et al., 2009). The drugs in each group were administered for three consecutive days. The body symptoms and mortality of each sub-group (10 mice) were recorded at 0, 4, 24, and 48 h post infection, correspondently.

### Counts of white blood cells (WBC) and pathological examination of lung

Cardiac blood samples were drawn from the mice infected with multi-drug resistant *A. baumannii* at 0, 4, 24, and 48 h post infection. The white blood cell counts were determined. The lungs were fixed in 40% formaldehyde, and paraffin-embedded sections were strained by hematoxylin and eosin. The lungs were subjected to pathological examination to evaluate morphology and inflammation (Hardy et al., 2009; Giladi et al., 2010). The mice in drug-treated groups (for three consecutive days) were sacrificed after therapy for 24, 48, and 72 h. WBC counts and lung pathological examination were conducted.

### Statistical analysis

The data were presented as mean ± standard deviation (SD). Differences between comparison groups were analyzed by Analysis of Variance (ANOVA) using SPSS19.0 software. *P* < 0.05 was considered significant difference.

## Results

### *In vitro* antibacterial activity of antimicrobial agents alone

The 101 multi-resistant strains tested in this study were completely resistant to piperacillin/tazobactam sodium, ceftazidime, levofloxacin, amikacin, fosfomycin sodium, chloramphenicol (Resistance rate: 100%), and had high resistance to imipenem/cilastatin sodium, cefoperazone/sulbactam, erythromycin (Resistance rate: >79%). By contrast, these strains had high sensitivity to rifampicin (Sensitivity: 79.2%), polymyxin B (Sensitivity: 78.2%) and minocycline (Sensitivity: 74.2%). The *in vitro* antimicrobial resistance values are listed in Table 2.

**Table 2:** **Thirteen normal antibiotics activity profile of multi-drug resistant *Acinetobacter baumannii***.

**Antibiotics**	**S/%**	**I/%**	**R/%**	**MIC range/(μg/ml)**	**MIC_50_**	**MIC_90_**
IMP/CS	14.3	2.2	83.5	1–256	32	64
TZP	-	-	100	64–4096	512	1024
SCF	-	8.8	91.2	4–256	32	128
CAZ	-	-	100	4–512	128	512
LEV	-	-	100	4–256	8	32
AMK	-	-	100	128–8192	2048	8192
E	15.8	5.0	79.2	2–256	64	256
RIF	79.2	20.8	-	0.5–32	4	16
TIG	98.0	2.0	-	0.125–2	0.25	0.5
PB	78.2	21.8	-	0.125–4	0.5	2
FOS	-	-	100	64–4096	512	4096
MNO	74.2	14.9	10.9	1–64	4	16
C	-	-	100	32-512	256	512

*PB, Polymyxin B; TIG, Tigecycline; MNO, Minocycline; FOS, Fosfomycin sodium; C, Chloramphenicol; RIF, Rifampicin; TZP, Piperacillin/tazobactam sodium; E, Erythromycin; IMP/CS, Imipenem/cilastatin sodium; AMK, Amikacin; SCF, Cefoperazone/sulbactam sodium; CAZ, Ceftazidime; LEV, Levofloxacin; S, I, R represents Susceptible, Intermediate and Resistant, respectively*.

### *In vitro* antibacterial activity of antimicrobial agents in combination

Chloramphenicol had no additive effects in combination with other antimicrobial agents, with the exception of polymyxin B. Similarly, fosfomycin had no additive effects with other antimicrobial agents, with the exception of erythromycin. However, all the remaining drug combinations showed either synergistic or additive effects (Table 3).

**Table 3:** **The fractional inhibitory concentration index (FICI) values of antibiotics combination**.

**Antibiotics**	**RIF**	**TZP**	**C**	**FOS**	**E**	**MNO**	**AMK**	**IMP/CS**	**PB**
PB	0.500	2.250	0.750	1.125	0.875	N.D.	N.D.	N.D.	N.D.
TIG	0.750	N.D.	1.125	1.125	0.750	0.625	0.875	N.D.	N.D.
MNO	0.750	N.D.	1.250	1.125	0.750	N.D.	0.750	N.D.	0.750
FOS	N.D.	N.D.	1.125	N.D.	0.875	N.D.	1.125	2.000	N.D.
C	N.D.	N.D.	N.D.	N.D.	N.D.	N.D.	N.D.	2.000	N.D.

*PB, Polymyxin B; TIG, Tigecycline; MNO, Minocycline; FOS, Fosfomycin sodium; C, Chloramphenicol; RIF, Rifampicin; TZP, Piperacillin/tazobactam sodium; E, Erythromycin; IMP/CS, Imipenem/cilastatin sodium; AMK, Amikacin; FICI was defined as FICI = MIC_A2_/MIC_A1_ + MIC_B2_/MIC_B1_. FICI index ≤0.5, 0.5–1, 1–4, >4 were defined as synergistic, addition, non-relation or antagonism, respectively. N.D., not done*.

### Results of time-kill curve experiments

**Figure 1:**
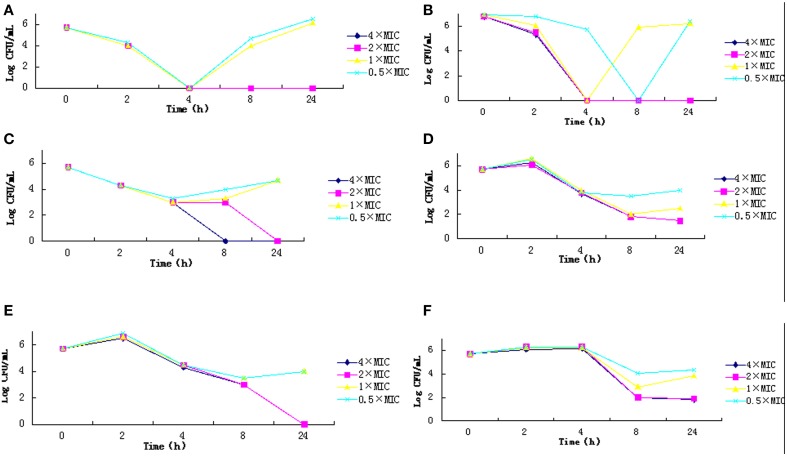
**Time-kill curve for the 6 types of antibiotics**. **(A)** C, Chloramphenicol; **(B)** PB, Polymyxin B; **(C)** FOS, Fosfomycin sodium; **(D)** TIG, Tigecycline; **(E)** RIF, Rifampicin; **(F)** MNO, Minocycline.

Chloramphenicol, polymyxin B and fosfomycin sodium at the concentrations of 4 × MIC or 2 × MIC significantly reduced the number of colonies within 4 h and completely eliminated the colonies within 8 h. However, at low concentrations (1 × MIC, 0.5 × MIC), they had no effects on bacterial growth. The strains proliferated during the first two hours with tigecycline and rifampicin. With minocycline treatment, the number of colonies increased within 4 h; if higher drug concentrations were used, they initially decreased, but displayed regrowth 8 h after treatment (Figure 1).

### *In vivo* antibacterial activity of antimicrobial agents

After ultrasonic atomization infection with *A. baumannii* for 0, 4, 24, and 48 h, WBCs in cardiac blood of model group were 1.31 ± 0.31, 1.84 ± 0.20, 2.73 ± 0.47, and 4.13 ± 1.10 (×10^9^/L), respectively. As shown by the data, the WBC counts increased significantly in a time-dependent manner. After drug treatment (consecutive 3 days), initiating from 0 h after infection by multi-drug resistant *A. baumannii*, WBCs were measured at 24, 48, and 72 h after drug treatments. WBCs in group D (MNO + RIF) were significantly higher than those in model group, group A (TIG), group B (PB) and group C (MNO + AMK) (*P* < 0.05). Compared with group C, WBCs in group A and group B were not significantly affected. After drug treatment (consecutive 3 days), initiating from 4 h post infection, WBCs in group C and group D were comparable (*P* > 0.05) at 24 h after drug treatment. However, compared with group A and group B, WBCs in group C and group D were significantly increased at 48 h after drug treatments. After drug treatment (consecutive 3 days) at 24 h after infection, WBCs in group C were significantly different from those in groups A, B, and D at 24 h after treatments. By contrast, WBCs in group D were comparable with the values in group A and group B at 24, 48, and 72 h after drug treatments. After drug treatment (consecutive 3 days), initiating from 48 post infection, WBCs in groups A, B, C, and D at 24, 48, and 72 h after drug treatments were significantly different from those in model group. However, WBCs in groups A, B, C, and D were comparable at 48 and 72 h after drug treatments. In addition, after treatments with single drug initiating from 24, 48 and 72 h post infection, there was no difference regarding WBCs at groups A and B (Table 4).

**Table 4:** **Mean values of WBC counts and standard deviations for different antibiotics treatment in several infection time (0, 4, 24, 48 h)**.

**Groups**	**24 h post-treatment**				**48 h post-treatment**				**72 h post-treatment**			
	**0 h^1a^**	**4 h^2a^**	**24 h^3a^**	**48 h^4a^**	**0 h^1b^**	**4 h^2b^**	**24 h^3b^**	**48 h^4b^**	**0 h^1c^**	**4 h^2c^**	**24 h^3c^**	**48 h^4c^**
A	1.90 ± 0.93	1.80 ± 0.62	3.10 ± 0.54	4.90 ± 0.56	2.30 ± 0.37	2.45 ± 0.67	2.97 ± 0.40	5.43 ± 0.13	2.05 ± 0.34	2.10 ± 0.36	2.72 ± 0.45	4.89 ± 0.24
B	1.90 ± 0.35	1.60 ± 0.41	3.20 ± 0.37	5.10 ± 0.12	1.65 ± 0.22	1.98 ± 0.15	2.95 ± 0.52	4.94 ± 0.62	2.34 ± 0.51	2.05 ± 0.24	2.89 ± 0.33	4.85 ± 0.32
C	1.70 ± 0.47	3.60 ± 0.69	4.26 ± 0.89	5.30 ± 0.53	2.00 ± 0.23	3.80 ± 0.33	4.60 ± 0.21	4.70 ± 0.18	2.02 ± 0.14	3.73 ± 0.28	4.50 ± 0.30	4.64 ± 0.41
D	4.60 ± 0.52	4.30 ± 0.25	4.03 ± 0.31	5.01 ± 0.40	5.20 ± 0.34	5.00 ± 0.13	4.30 ± 0.45	4.62 ± 0.69	4.90 ± 0.31	4.30 ± 0.40	4.10 ± 0.23	4.57 ± 0.37

*Group A, Tigecycline (TIG); Group B, Polymyxin B (PB); Group C, Minocycline +Amikacin (MNO+AMK); Group D, Minocycline + Rifampicin (MNO+RIF)*.

*P-values come from multiple comparisons for different antibiotics treatment group in the same infection time*.

*P_AB_ represents the P-value of multiple comparisons between Group A and Group B; P_AC_ represents the P-value of multiple comparisons between Group A and Group C*.

*P_AD_ represents the P-value of multiple comparisons between Group A and Group D; P_BC_ represents the P-value of multiple comparisons between Group B and Group C*.

*P_BD_ represents the P-value of multiple comparisons between Group B and Group D; P_CD_ represents the P-value of multiple comparisons between Group C and Group D*.

*1a: P_AB_ = *0.283*; P_AC_ = *0.381*; P_AD_ = *0.018*; P_BC_ = *0.834*; P_BD_ = *0.028*; P_CD_ = *0.040**.

*2a: P_AB_ = *0.927*; P_AC_ = *0.154*; P_AD_ = *0.009*; P_BC_ = *0.178*; P_BD_ = *0.010*; P_CD_ = *0.125**.

*3a: P_AB_ = *0.727*; P_AC_ = *0.002*; P_AD_ = *0.605*; P_BC_ = *0.001*; P_BD_ = *0.392*; P_CD_ = *0.005**.

*4a: P_AB_ = *0.251*; P_AC_ = *0.004*; P_AD_ = *0.013*; P_BC_ = *0.031*; P_BD_ = *0.011*; P_CD_ = *0.024**.

*1b: P_AB_ = *0.055*; P_AC_ = *0.191*; P_AD_ = *0.000*; P_BC_ = *0.061*; P_BD_ = *0.000*; P_CD_ = *0.000**.

*2b: P_AB_ = *0.083*; P_AC_ = *0.000*; P_AD_ = *0.000*; P_BC_ = *0.000*; P_BD_ = *0.000*; P_CD_ = *0.001**.

*3b: P_AB_ = *0.946*; P_AC_ = *0.028*; P_AD_ = *0.058*; P_BC_ = *0.032*; P_BD_ = *0.065*; P_CD_ = *0.069**.

*4b: P_AB_ = *0.408*; P_AC_ = *0.245*; P_AD_ = *0.191*; P_BC_ = *0.719*; P_BD_ = *0.605*; P_CD_ = *0.873**.

*1c: P_AB_ = *0.296*; P_AC_ = *0.971*; P_AD_ = *0.000*; P_BC_ = *0.312*; P_BD_ = *0.000*; P_CD_ = *0.000**.

*2c: P_AB_ = *0.841*; P_AC_ = *0.000*; P_AD_ = *0.000*; P_BC_ = *0.000*; P_BD_ = *0.000*; P_CD_ = *0.039**.

*3c: P_AB_ = *0.800*; P_AC_ = *0.022*; P_AD_ = *0.062*; P_BC_ = *0.034*; P_BD_ = *0.097*; P_CD_ = *0.561**.

*4c: P_AB_ = *0.931*; P_AC_ = *0.635*; P_AD_ = *0.547*; P_BC_ = *0.697*; P_BD_ = *0.605*; P_CD_ = *0.897**.

### Changes of vital signs and mortality rate

We established a new lung infection model by ultrasonic atomization. Immediately after infection, there was no mortality in any group. In the early stage after infection (0–4 h), a few mice in the model group presented with symptoms such as shortness of breath and loss of activity. However, after more prolonged infection, mice mortality rates gradually increased to 80%. Except for group D, some mice of other three groups died in the first 4 h after infection. However, at longer infection times (24 or 48 h post-infection), the medication groups showed increased mortality. The mortality rates in drug combination groups (groups C and D) were much lower than in groups A and B. The mortality rates in medication groups at all time points were lower than the corresponding values in the model group (Table 5). These results suggested that the drug combinations were much more effective than single drug treatment.

**Table 5:** **Effect on mortality rates [% (n/N)] at different infected time for each group**.

**Groups**	**0 h**	**4 h**	**24 h**	**48 h**
Model	0.0 (0/10)	30.0 (3/10)	60.0 (6/10)	80.0 (8/10)
A	0.0 (0/10)	10.0 (1/10)	10.0 (1/10)	50.0 (5/10)
B	0.0 (0/10)	10.0 (1/10)	30.0 (3/10)	60.0 (6/10)
C	0.0 (0/10)	10.0 (1/10)	20.0 (2/10)	40.0 (4/10)
D	0.0 (0/10)	0.0 (0/10)	10.0 (1/10)	50.0 (5/10)

*Group A, Tigecycline (TIG); Group B, Polymyxin B (PB); Group C, Minocycline + Amikacin (MNO+AMK); Group D, Minocycline + Rifampicin (MNO+RIF)*.

### Pathological changes in the pneumonia model and after drug treatment

**Figure 2:**
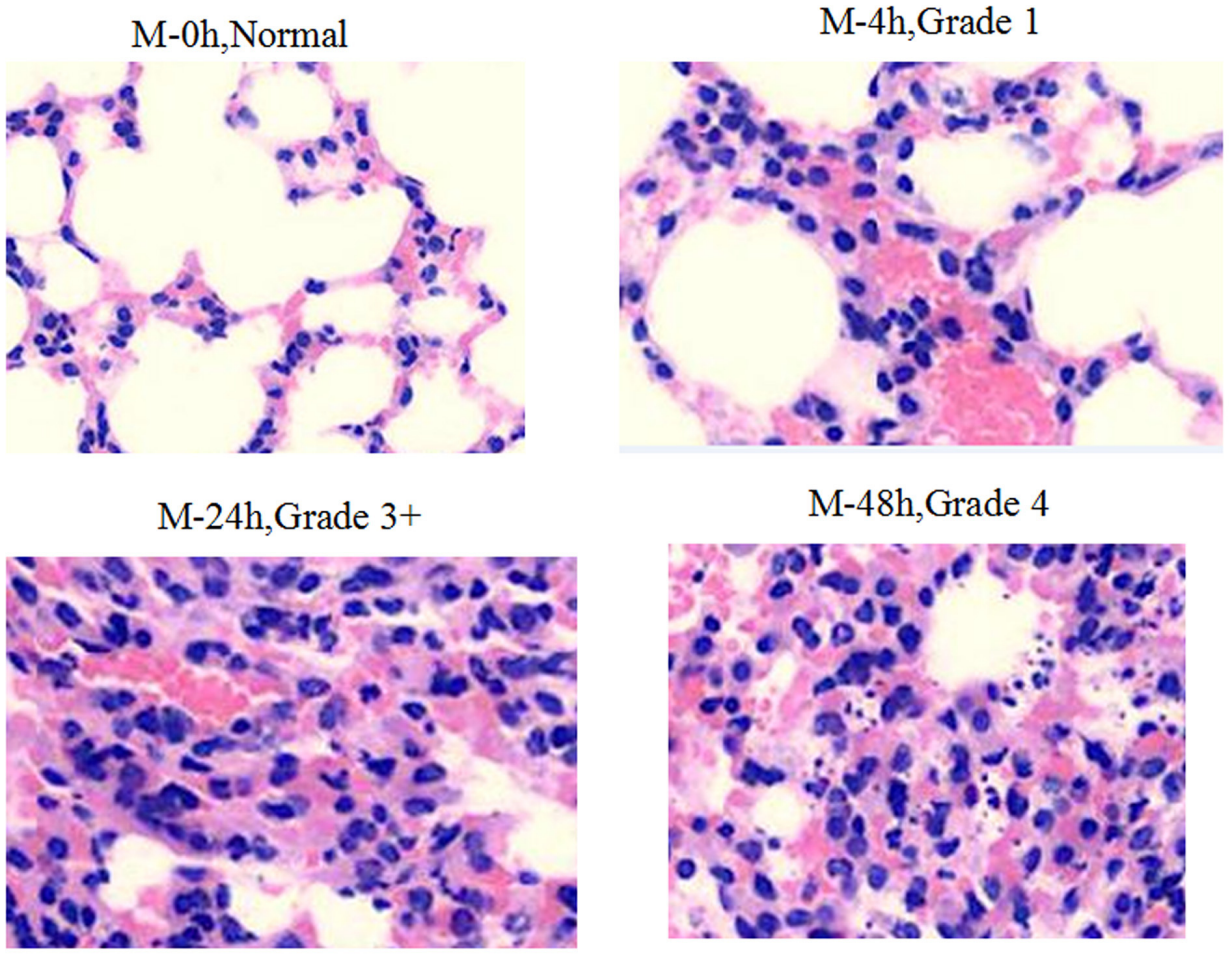
**Pathological changes of Model group (hematoxylin-eosin staining, ×200)**.

In the model group (Figure 2) at baseline the morphology of lung tissue was normal, with regular structure, no interstitial inflammation, slightly dilated blood vessels and no infiltration of inflammatory cells. Four hours after infection, there was a lung tissue inflammation reaction composed mostly of lymphocytes (Grade 1: the amount of inflammatory cell infiltration was less than 20%). Twenty-four hours after infection, the pulmonary infiltration of lymphocytes was greatly increased (Grade 2: the amount of inflammatory cell infiltration was between 20 and 40%). Meanwhile, a small number of neutrophils and macrophages were observed. Between 24 and 48 h after infection, a large number of inflammatory cells were found (mainly neutrophils, Grade 3: the amount of inflammatory cell infiltration was 41–80%); 48 h after infection, there were severe inflammation findings, including vascular dilation, congestion with hemorrhage, neutrophils, lymphocyte and macrophage infiltration in bronchial and alveolar (Grade 4: the amount of inflammatory cell infiltration was >80%), a significant dilation of blood vessels, congestion with hemorrhage, collapse of part of the alveolar structure and increased visible bacterial colonies in alveolar abscesses.

**Figure 3:**
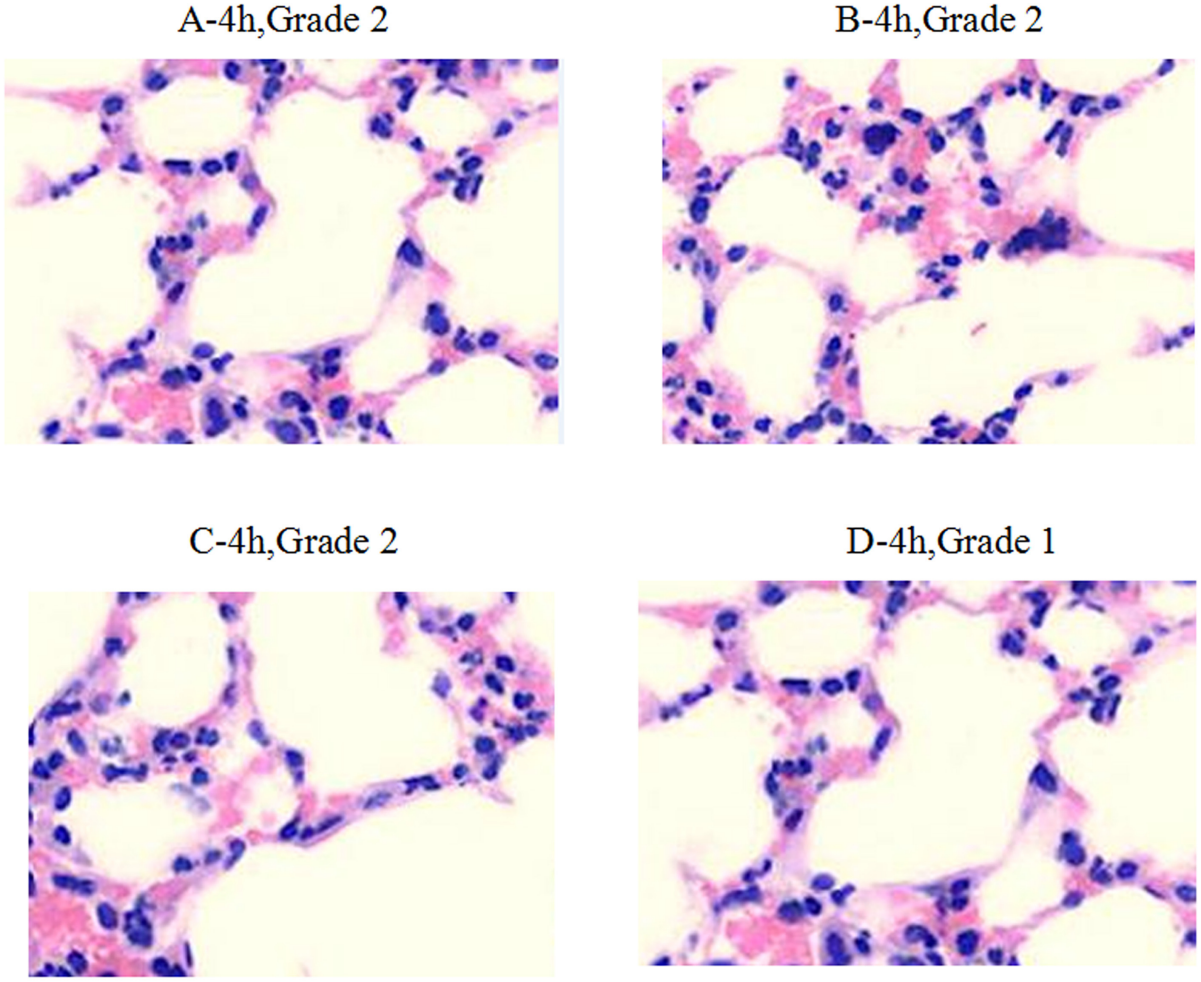
**Pathological changes of treatment groups at 4 h post infection (hematoxylin-eosin staining, ×200)**.

**Figure 4:**
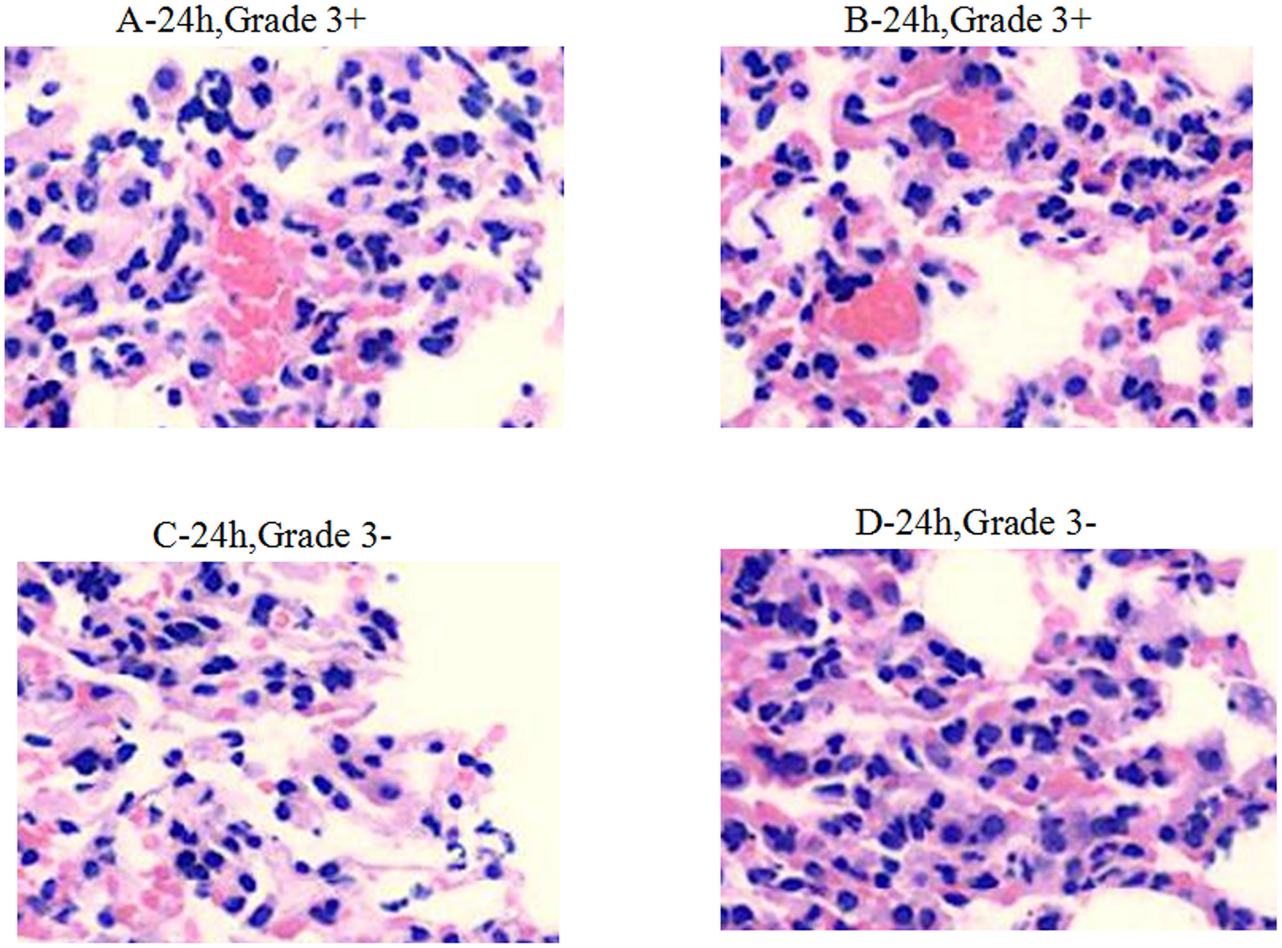
**Pathological changes of treatment groups at 24 h post infection (hematoxylin-eosin staining, ×200)**.

**Figure 5:**
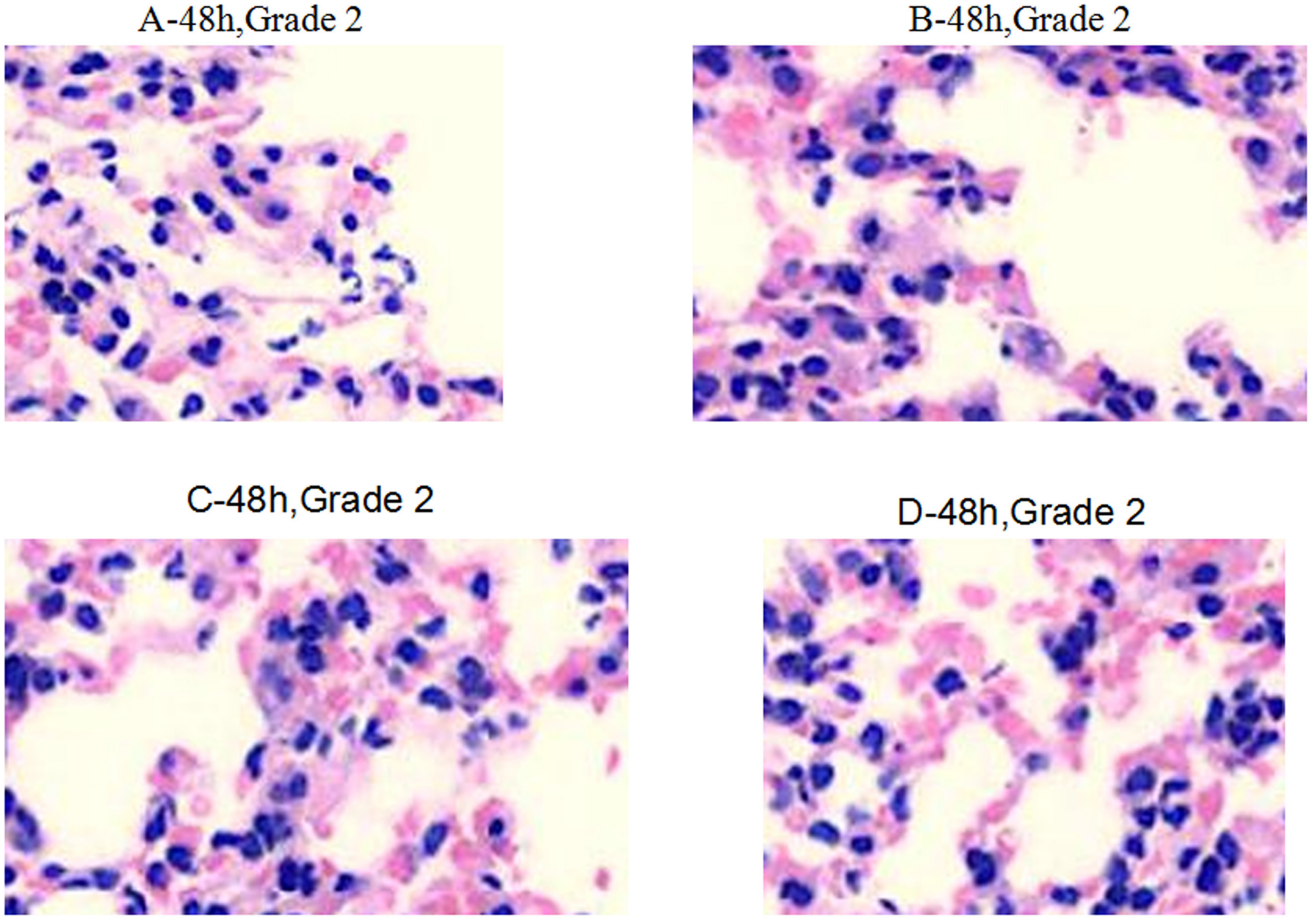
**Pathological changes of treatment groups at 48 h post infection (hematoxylin-eosin staining, ×200)**.

Within 4 h after infection, the medication in groups A, B, C, and D caused decreased inflammatory infiltration (Grade 2). Inflammatory cell infiltration in group D was much lower than in other groups (Grade 1) (Figure 3). Within 24–48 h after infection, obvious inflammation infiltration and local hemorrhage were observed in groups A and B (Grade 3+), and obvious inflammatory reactions were observed in groups C and D (Grade 3−) without lung tissue disintegration (Figure 4). After consecutive 3 days of anti-infective therapy in groups A, B, C, and D, lung tissue inflammation in each group gradually recovered with clear structures (Grade 2) (Figure 5).

## Discussion

*A. baumannii* is becoming an important pathogen with the ability of causing nosocomial infections (Ozturk et al., 2014). With the increasingly widespread use of antimicrobial drugs, *A. baumannii* has developed resistance to several drugs, with the emergence of multi-drug resistant strains (Bassetti et al., 2011). In this study, we found that *A. baumannii* displayed low sensitivities to carbon penicillins, cephalosporins, and aminoglycosides. The MIC_90_ of these drugs were more than 64 μg/ml. The loss of sensitivity to those drugs might be due to the wide use of antibiotics in the clinical practice. However, *A. baumannii* was still sensitive to tigecycline (MIC_90_: 0.5 μg/ml), polymyxin B (MIC_90_: 2 μg/ml), minocycline (MIC_90_: 16 μg/ml) and rifampicin (MIC_90_: 16 μg/ml). Meanwhile, the combinational experiments showed that tigecycline, polymyxin B and rifampicin displayed synergistic or additive effect when combined with other drugs. The FICI in all the combination groups in this study were between 0.5 and 2.25.

Tigecycline is a new type of glycyl prostacyclin antimicrobial agent, which inhibits bacterial protein translation and produces antibacterial effects which are modulated by different factors, including resistance-nodulation-cell division (RND)-type transporters and other efflux pumps (Sun et al., 2013). Polymyxin B inhibits bacterial growth by increasing membrane permeability (Liu et al., 2014). However, because of its nephrotoxicity and neurotoxicity, this drug is preferably used in combination in the clinical practice, allowing a dose reduction to alleviate the toxic effects. Minocycline achieves its bactericidal effects through protein synthesis inhibition (Rumbo et al., 2013). Because of dose-dependent effects on the gastrointestinal tract and the vestibular system, minocycline alone might not be suitable for anti-infection of multi-drug resistant *A. baumannii*. In combination with other antimicrobial agents, minocycline has been able to achieve significant anti-infective effect (Zhang et al., 2013).

The antibacterial activity *in vitro* does not necessarily reflect the activity *in vivo*. Hence, a stable and effective animal model is required to evaluate novel therapeutic approaches and clearly identify bacterial virulence factors (McConnell et al., 2013). Mice (such as C57BL/6, BALB/c, and A/J, etc.) have a predisposition to a variety of pathogens, and mice models are frequently used to study antimicrobial infection and related pathogenesis. BALB/c line (inbred) mice are susceptible to pneumonia. In this study BALB/c mice were used for an animal model of bacterial pneumonia (Chiang et al., 2013). The ultrasonic atomization method was used to establish the pneumonia model. Before the establishment of the model, methotrexate was used to reduce mouse white blood cells, resulting in immunodeficient mice. After atomization, the mortality and WBCs gradually increased with infection time. Several inflammatory cells (mainly neutrophils) were observed in the lungs 24–48 h after infection. In addition, clinical manifestations of lung inflammation were detected in model mice, including shortness of breath, weight loss, appetite loss, reduced activity. These symptoms mimicked the clinical presentation of pneumonia, which confirmed the validity of our model. This allowed us to screen novel therapeutic strategies for multi-drug resistant *A. baumannii* pulmonary infection, as well as to investigate the infection mechanisms.

After successful establishment of the mouse pneumonia model, the efficacy of drug treatment and pathological changes in lung tissue were analyzed. According to *in vitro* susceptibility test, polymyxin B and tigecycline were effective in inhibiting multi-drug resistant *A. baumannii*, and the combinations (minocycline + amikacin and minocycline + rifampicin) had synergistic antibiotic effects. We tried to confirm the *in vitro* data using our pneumonia model. In this study, within 4 h after infection, WBC counts in treatment group D was higher than in groups A, B, and C. These data indicated that minocycline and rifampicin had synergistic effect in the early stage after infection. Regarding the time-kill curve experiments, proliferation of bacteria within 4 h was found in mice treated with minocycline or rifampicin, although the number of colonies gradually decreased with the duration of treatment time. In contrast, the minocycline and rifampicin combination was largely effective in mice within 4 h after infection. The possible mechanisms might be due to the inhibition of protein synthesis by minocycline and RNA translation at early phase by rifampicin (Jamal et al., 2014). However, within 24–48 h after infection, the inhibition effect in group C became clear and WBC counts were significantly higher compared with that in group D. Amikacin inhibits bacterial growth by increasing membrane permeability at logarithmic growth, leading to release of the functional substance. On the contrary, minocycline functions through inhibiting protein synthesis. The combination of these two drugs has synergistic or additive effect (Cunha, 2006).

Song et al also reported that the combination of two drugs among polymyxin B, imipenem and rifampicin was effective against multi-drug resistant *A. baumannii* (Song et al., 2009). In a late phase after infection (24–48 h), the medication group had obvious effects, when compared with the model group. Meanwhile, according to the time-kill curve experimental data, six high concentrations (4 × MIC, 2 × MIC) of antimicrobial agents reduced the amount of the colony count more than 3 log_10_ within 24 h, suggesting that an increase in the concentration of antibacterial drug to some extent, can achieve a good bactericidal effect *in vitro*. However, in time-kill curve experiments, bacteria treated with the studied agents at low concentrations displayed an activity of regrowth after 24 h. The degradation of tigecycline might contribute to the loss of drug effect. For other antimicrobial agents, the regrowth after 24 h may be due to the pharmaceutical gradual failure.

The pathological changes after drug treatments also suggested that drugs in combination had synergistic or additive effects compared with the same drugs administered as single agents. Importantly, minocycline and rifampicin combination had a prominent effect in mice within 4 h after infection, while amikacin and minocycline combination had synergistic or additive effect 24 h after infection.

Minocycline is a well-characterized and safe second-line antimicrobial drug. Both *in vivo* and *in vitro* studies indicated that minocycline has antibacterial effect on multi-drug resistant *A. baumannii*. Also, thanks to its low cost, minocycline can play an important role in the treatment of multi-drug resistant *A. baumannii*. However, this drug should be avoided as single agent. The combination with other antibacterial drugs not only reduces the doses of single drugs used, but also lowers the risk of bacterial resistance (Zavascki et al., 2010). On the other side, rifampicin and amikacin are safe, effective, economical drugs, which are widely used in the clinic. Therefore, either minocycline + amikacin or minocycline + rifampicin not only can reduce the treatment cost, but can also increase the inhibition activity against multi-drug resistant *A. baumannii*.

Although multi-drug resistant *A. baumannii* was sensitive to tigecycline and polymyxin B in *in vitro* experiments, as showed in the time-kill curve experiments, the effects of these drugs *in vivo* are not certain. These data further confirmed the disagreement of *in vivo* and *in vitro* effects in antibacterial activity. The following reasons might explain this discrepancy. First, based on pharmacokinetic analysis, the drug half-life *in vivo* affects the absorption and distribution of drugs; second, different routes of administration directly affect drug absorption, distribution, metabolism and excretion, thus modifying the concentration of drug in the body and the behavior over time, ultimately affecting drug efficacy; third, relevant uncontrollable factors affect animal experiments; fourth, *A. baumannii* can be divided into mucinous and non-mucinous types. The mucinous type can easily form colonies in the lung and recovery is difficult following treatment (Neonakis et al., 2014). Therefore, it is necessary to establish an animal model for *in vivo* screening of anti-bacterial drugs.

In conclusion, this study demonstrated that tigecycline and polymyxin B were highly sensitive to multi-drug resistant *A. baumannii* in an *in vitro* susceptibility test and, when combined with other drugs, they can produce synergistic or additive effects. *In vivo* experimental data indicated that minocycline in combination with either rifampicin or amikacin was more effective against multi-drug resistant *A. baumannii* than tigecycline or polymyxin B alone. In addition, the ultrasonic atomization lung infection model can simulate the entire processes of clinical infectious pneumonia. This model can be used to explore the mechanisms and to screen new drugs against multi-drug resistant *A. baumannii* infection.

### Conflict of interest statement

The authors declare that the research was conducted in the absence of any commercial or financial relationships that could be construed as a potential conflict of interest.
